# A Novel Software and Method for the Efficient Development of Polymorphic SSR Loci Based on Transcriptome Data

**DOI:** 10.3390/genes10110917

**Published:** 2019-11-11

**Authors:** Ruizheng Tian, Cunhuan Zhang, Yixiao Huang, Xin Guo, Maohua Chen

**Affiliations:** Northwest A&F University, State Key Laboratory of Crop Stress Biology for Arid Areas, Key Laboratory of Integrated Pest Management on Crops in Northwestern Loess Plateau, Ministry of Agriculture and Rural Affairs, Yangling 712100, China; ianruizheng@nwafu.edu.cn (R.T.); zhangch@wh.iov.cn (C.Z.); yixiaohuang@nwafu.edu.cn (Y.H.); xinguo@nwafu.edu.cn (X.G.)

**Keywords:** SSR, transcriptomes, polymorphic, method

## Abstract

Traditional methods for developing polymorphic microsatellite loci without reference sequences are time-consuming and labor-intensive, and the polymorphisms of simple sequence repeat (SSR) loci developed from expressed sequence tag (EST) databases are generally poor. To address this issue, in this study, we developed a new software (PSSRdt) and established an effective method for directly obtaining polymorphism details of SSR loci by analyzing diverse transcriptome data. The new method includes three steps, raw data processing, PSSRdt application, and loci extraction and verification. To test the practicality of the method, we successfully obtained 1940 potential polymorphic SSRs from the transcript dataset combined with 44 pea aphid transcriptomes. Fifty-two SSR loci obtained by the new method were selected for validating the polymorphic characteristics by genotyping in pea aphid individuals. The results showed that over 92% of SSR loci were polymorphic and 73.1% of loci were highly polymorphic. Our new software and method provide an innovative approach to microsatellite development based on RNA-seq data, and open a new path for the rapid mining of numerous loci with polymorphism to add to the body of research on microsatellites.

## 1. Introduction

The increasing progress of next generation sequencing (NGS) has promoted the explosion of transcriptome data, providing large-scale essential data for the application of molecular markers [1,2,3]. Microsatellites, also called simple sequence repeats (SSRs) or short tandem repeats (STRs), are among the most popular markers, and have been widely used in the analysis of population genetics due to such advantages as wide distribution, high polymorphism, and satisfactory repeatability [4,5,6,7,8]. The applications of SSR markers can obtain abundant and reliable experimental data based on the hypermutation of SSRs. However, traditional methods of polymorphic SSR isolation and characterization without reference sequences are expensive, time-consuming and labor-intensive [9,10]. Many researchers have attempted to optimize and streamline microsatellite experiments [11,12,13,14,15]. For instance, the application of multiplex PCR can significantly reduce the time and cost of SSR genotyping [11], and the success rate of tests can be increased by referring to the propositions concerning the primer design of SSR loci [12]. In addition, to reduce the risk of failure, SSR loci over a certain repeat time were presumed to be polymorphic for follow-up research, resulting in large loci with fewer repeats being overlooked [16].

Even though SSR loci obtained from whole genome data are generally polymorphic [17,18], the genomes of many species have not been sequenced. Compared to genomic-SSR analysis, the development of SSRs based on RNA-seq data has also become a mature and commonly employed method due to its low cost [19,20,21,22,23]. However, not all of the microsatellites tend to mutate, many SSR loci may not show polymorphisms among individuals, especially the loci in the gene encoding region excavated from transcriptome data, as a result, SSR mining using this traditional method is not always efficient [19,23,24,25,26,27]. Additionally, although some studies obtained many polymorphic SSR loci from transcriptome data, few of these loci showed high polymorphism [19,28,29,30]. For instances, during the SSR development in *Sander lucioperca* based on its RNA-seq data, Han et al. (2016) found that 18% of SSR loci were polymorphic and only one locus (1%) was highly polymorphic [28]. Li et al. (2017) found 15 polymorphic SSR loci and five highly polymorphic loci in the 55 test loci using the *Casuarina equisetifolia* transcriptome [29]. The indel analysis of whole genome re-sequencing can provide large scale data to obtain microsatellite mutation information [31,32,33,34]. However, due to the high costs, genome re-sequencing is not always available or unpractical. Additionally, because the software for indel analysis, such as Samtools or GATK [35], is not specifically designed for SSR analysis, the detection of SSRs using this type of program might have a higher error rate and the whole process requires some additional fresh script. Similar situations are encountered in transcriptome dataset analysis. Some indel analysis software can be used for RNA-seq data [35,36,37], none of which has been optimized for SSR detection alone.

In the present study, a new method for simplifying polymorphic SSR site screening using RNA-seq data is proposed. In brief, the concise method primarily consists of three stages: raw data processing, software development and implementation, and the extraction and checking of sequences. A new Perl-based open-source software developed in this study is the key component for the task, which efficiently processes the dataset of multiple transcriptomes. Providing a clear experimental range for polymorphic site mining, the new software can obtain numerous unverified polymorphic SSR loci. Additionally, the software can reflect the polymorphism details of most SSR loci, meaning that the SSR loci with high polymorphism can be further selected and many SSRs with few repeats will not be neglected. However, the method is generally unable to be used for species with transcriptomes less than five. We verify the practicability of the method in pea aphid (*Acyrthosiphon pisum*) individuals by genotyping.

## 2. Materials and Methods

### 2.1. Data Collection

The RNA-seq of pea aphids were used in this study. A total of 44 pea aphid transcriptomes were downloaded from National Center for Biotechnology Information (NCBI) SRA database (https://www.ncbi.nlm.nih.gov/sra/). The RNA-seq data was submitted by nine different institutions including the University of Arizona [38], University of Nebraska-Lincoln, Baylor College of Medicine [39], Centro Nacional de Análisis Genómico, Cornell University, Yale University [40], French National Institute for Agricultural Research, Gene Expression Omnibus [41], and National Institute for Basic Biology (Table 1).

### 2.2. Development of a New Software

To establish a novel method for screening polymorphic SSR loci using multiple transcriptomes, we first developed one software (Polymorphic SSR digging tool, PSSRdt, publicly available at https://github.com/PSSRdt/program). This program is based on Perl and available in the input files in FASTA format, which is compatible with multiple systems including Linux and Windows. The usage of PSSRdt is consistent with the regular Perl scripts and is also listed in the software manual. The program primarily depends on the data primitiveness of transcripts assembled using the de novo assembly method. PSSRdt first seeks out SSRs from the input file. The criteria of SSR detection refer to microsatellite searching program-MISA (available online at http://pgrc.ipk-gatersleben.de/misa/misa.html) (Table S1). Unlike some specialized software for SSR identification such as MISA or SSRLocator [42], this program does not need to distinguish whether SSRs are perfect (with single simple repeats) or imperfect [43]. SSR motifs screened with their flanking sequences are then recorded as the hash ‘keys’ and will be assessed as one SSR locus for the moment, the repeat number of which will be logged as their hash ‘values’. The various SSR loci excavated from the input file combined by multiple transcriptomes can be classified and their repeat details will be quickly saved and listed. Thus, if only a few RNA-seq data are available, such as one to five, the digitals in ‘values’ will be not enough for analysis. In addition, the length of each flanking sequence is determined by the judgment of the users according to thespecific data assembled quality (usually over eight to improve the accuracy). PSSRdt needs to call the scripts in Bioperl (available online at https://bioperl.org/). Users should first ensure the setup success of Bioperl modules, where the common installation methods of the modules are listed in the software manual.

### 2.3. Screening and Verifying of SSR Obtained by PSSRdt

**Figure 1:**
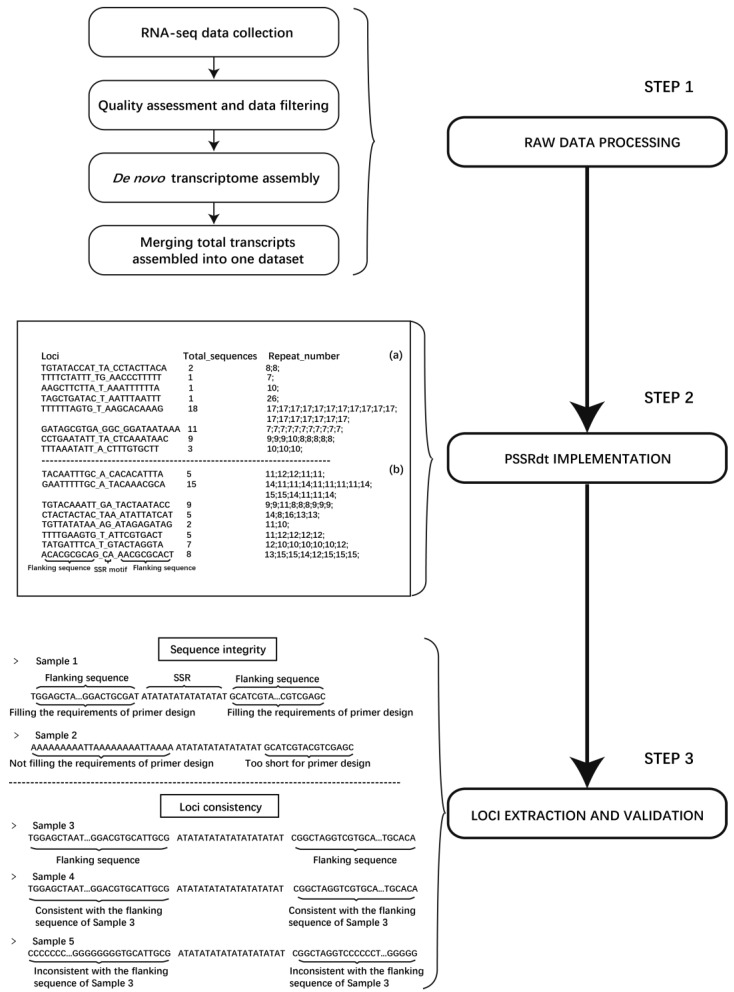
Flow diagram of the method for polymorphic SSR loci mining. Step 1 indicates the procedure of RNA-seq raw data processing. Two examples represent the characteristics of the two output files generated by PSSRdt on the left of Step 2. (**a**) and (**b**) correspondingly represent total screened SSR loci and potential polymorphic loci. Two main validation items for the information check of potential polymorphic SSR are listed in Step 3. The flanking sequences in Sample 1 fill the requirements of primer design, while those in Sample 2 do not. The flanking sequences in Sample 4 are consistent with the corresponding flanking sequences in Sample 3, which are identified as the same SSR locus, while Sample 5 is not.

After running PSSRdt, users will obtain two output files containing the details of total detected SSRs and unverified polymorphic ones respectively (Figure 1 Step 2). Both files consist of three column contents. Each row represents the details of all detected SSRs in the assumed same SSR locus, which includes, in turn, the SSR motif with its flanking sequences of that locus, the sum of all SSRs in the locus and the repeat number of each SSR in that locus. The highly variable numbers of repeat motifs shown in the third column indicate the site with relatively high polymorphism. Therefore, users could select the loci with many different repeat numbers to improve the efficiency of subsequent tests. Based on SSRs with the flanking sequences shown in ‘FindStr.result’, users can quickly verify the correctness of polymorphism information and obtain the complete sequences of these loci using a string search function in any text editor. Meanwhile, users could check SSR loci carefully and extract the applicable sites in the same way. The most important items the users need to check are listed in Step 3 of Figure 1, including estimating whether the length of flanking sequences of SSR motifs meets criteria for primer design (the flanking sequences of Sample 1 could fill the primer design, while Sample 2 could not) and the consistency of all sequences in assumed same loci (the flanking sequences in Sample 4 are consistent with corresponding sequences in Sample 3, which are identified as the same SSR locus. Sample 5 and 3 are not the same locus).

### 2.4. Overall Flow of the Novel Method

A novel method was constructed to efficiently excavate potential polymorphic microsatellite loci using multiple transcriptomes, which was divided into three steps (Figure 1). First, in Step 1, different transcriptomes of an organism are collected and downloaded. After quality control and data filtering of raw data, de novo assembly software is applied to the high-quality clean reads. All transcripts assembled in FASTA format are then merged into one dataset, which will be calculated by PSSRdt in Step 2. Two output files will be immediately generated, including the details of all excavated SSR loci (called ‘FindStr.result.detail’) and the potential polymorphic sites (called ‘FindStr.result’). Users can select some potential polymorphic SSR loci from the ‘FindStr.result’ file. In Step 3, SSRs with complete flanking sequences can be simply and visually extracted using the details of these SSR loci by the text editors and the verification of sequence information accuracy can be performed simultaneously.

### 2.5. Experimental Validation

#### 2.5.1. RNA-Seq Data Assembly

To intuitively present and test the method, the experiments were conducted with pea aphids as samples. The raw reads of pea aphid transcriptomes were filtered to generate clean reads by removing adapter sequences, low-quality reads (quality scores < 30), reads with unknown bases ‘N’ and < 30 bp reads. All raw reads were assembled into transcripts using Trinity [44], the short reads assembling program. We integrated all 44 transcriptomes assembled into one dataset after *de novo* assembly. The ‘cat’ command on Linux was used to realize this step. The dataset was then computed by PSSRdt (parameter: 10) as an input file. Sublime Text 3 was performed to check the SSR details generated by PSSRdt and extract 100–300 bp sequences containing SSRs (each flanking sequence ≥ 50 bp).

#### 2.5.2. Samples and Primers

Pea aphid individuals were used for validation of the SSR polymorphism. These individuals are randomly taken from five populations originated from different locations and were fed by dactylethrae with broad bean plants in phytotron. The cultivation conditions were set to temperature 24 ± 1 °C, relative humidity 60% ± 10%, photoperiod L:D = 16 h:8 h.

We tested 52 SSR loci with high polymorphisms in the ‘FindStr.result’ file for validation by genotyping. SSR primers were designed by PRIMER3 [45] and the options for primer design refer to the following: (1) primer lengths ranging from 18 to 27 bp; (2) product sizes are 100–300 bp; (3) melting temperature (Tm) is 57 °C to 63 °C and the difference of Tm between forward and reverse primers < 2 °C; (4) GC content 40–60%, with an optimal value of 50%. If no primers were found with these options, the parameter range was adjusted: Tm 45–63 °C; the difference between forward and reverse primers < 5 °C; GC content 30–70%. The study referred to the microsatellite PCR fragment fluorescent labeling method [46]. Three PCR primers are needed to amplify each microsatellite locus by this method. The first primer was the 5′-end of the forward primers (F) adding M13 forward primer (5’-TGTAAAACGACGGCCAGT-3’); the second primer was the reverse primer (R) without any modification; and the third primer was a M13 forward primer labeled by 6-carboxy-fluorescine (FAM) at its 5’-end.

#### 2.5.3. PCR Amplification and Statistical Analysis

We extracted genomic DNA from individual *A. pisum* sample using the Easy Pure Genomic DNA kit (TransGen Biotech Co., Ltd., Beijing, China). Five individuals were randomly selected from each of the five populations for DNA extraction. Because of the small body size, the DNA extracted from a single pea aphid sample can only be used for the verification of 15 pairs of primers. Therefore, after the DNA was used up, we took another five individuals from each of the population for the next 15 primers, amounting to 25 pea aphid individuals from each population were used for validation of the 52 SSR loci selected. The DNA extracted was tested by 1.0% agarose gel electrophoresis to estimate the quality. Each 25 μL PCR included 1.5 μL pea aphid genomic DNA (the concentration of the primers 10–30 ng/μL), 12.5 μL of TransGen Biotech Taq MasterMix, 0.5 μL forward primer (10 μM), 2.0 μL reverse primer (10 μM), M13 primer 2.0 ul (10 μM). The PCR amplification conditions for microsatellite loci were as follows: DNA initial denaturation at 94 °C for 2 min; 30 cycles of 94 °C for 20 s, annealing temperature of specific primer for 20 s, 72 °C for 20 s; 8 cycles of 94 °C for 30 s, 53 °C for 45 s (special annealing temperature of primer M13), 72 °C for 45 s, and a final step 72 °C for 10 min.

The PCR products of microsatellite DNA were detected by 2% agarose gel electrophoresis. The products containing target bands were sent to Sangon Biotech for microsatellite genotyping detection. The PCR products with fluorescent labels were taken for fluorescence detection using capillary electrophoresis method in an ABI3730XL DNA automated analyzer (Applied Biosystems, Foster City, CA, USA). The obtained peak electrophoregrams were converted into the fragment length of amplified products using GENEMAPER v4.0 software (Applied Biosystems, Foster City, CA, USA). Lastly, the number of alleles (Na), polymorphism information content (PIC) and other analyses for evaluating polymorphism of SSRs were conducted by PowerMarker v3.25 [47].

## 3. Results

### 3.1. Transcriptome Data Assembly and Microsatellite Detection

**Figure 2:**
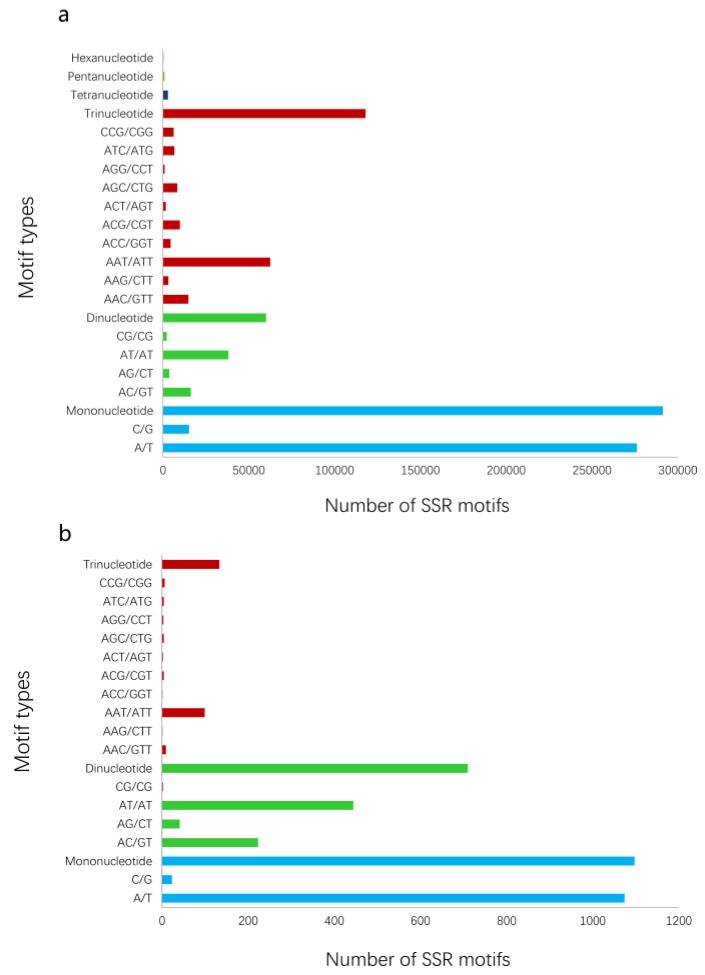
The number of SSRs in *A. pisum* based on motif types. (**a**) The number of total types of SSR motifs in the transcript dataset. (**b**) The number of SSR motifs with potential polymorphism analyzed by PSSRdt.

To intuitively present the overall processes of the proposed method, all results of verification experiments on pea aphids are provided. A total of 44 representative pea aphid transcriptomes were downloaded from the NCBI SRA public database (Table 1). Nine institutions submitted the RNA-seq data. Using the data, we obtained 3,387,696 to 126,263,308 raw reads, and 2,829,317 to 108,646,204 high quality clean reads were then generated by data filtering for next *de novo* assembly (Table S2). Running Trinity, the various pea aphid transcriptomes were assembled into 7634 to 68,321 transcripts and the total sizes of sequences ranged from 2,076,016 bp to 42,402,758 bp (Table 1). SSR analysis of transcriptomes using MISA is also exhibited in Table 1. In addition, the number of trinucleotide repeat motifs in all transcriptomes exceeded the dinucleotide repeats but were less than the mononucleotides (Figure 2a). SSR motifs of AT/AT, AG/CT, and AAT/ATT were dominant in dinucleotide and trinucleotide microsatellites.

### 3.2. PSSRdt Application

The dataset (3137 Mb, millions of base pairs) merged by 44 RNA-seq assembled data contained 1,089,298 transcripts. PSSRdt were executed on Intel i7-4770 1600 MHz with 4 Gb RAM, running on Windows 7. The transcript set in FASTA format was processed in 14 min and produced two documents, which included a total of 16,384 SSR loci (Supplementary File S1) and 1940 potential polymorphic loci (Supplementary File S2) respectively. The analysis of the ‘FindStr.result’ file in the Supplementary File 2 revealed that the mononucleotide repeats were predominant, reaching 1097 (56.55%). There were 710 (36.60%) more dinucleotide SSR motifs more than trinucleotide SSRs (133, 6.86%) (Figure 2b), which was not inconsistent with the quantity relationship shown in Figure 2a. The highest proportions in mononucleotide and dinucleotide repeats were A/T (1074, 55.33%) and AT/AT (444, 22.87%) respectively. Among trinucleotide SSRs, the AAT/ATT type was most abundant (108, 5.56%).

### 3.3. Primer Design and SSR Loci Validation

**Figure 3:**
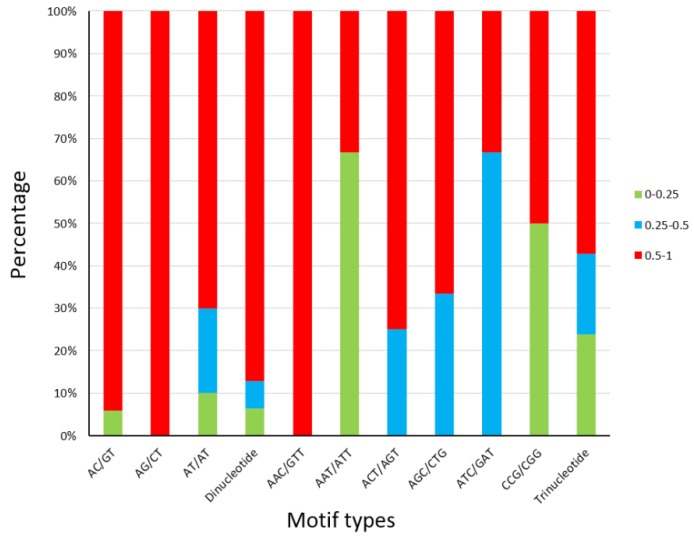
Polymorphism information content (PIC) details of SSR loci tested on *A. pisum*. The percentages of different PIC values in 9 types of SSR motifs, dinucleotide microsatellites, and trinucleotide microsatellites were visualized by three colors.

Primer sequences are showed in Table S3. The statistics of polymorphism for the 52 SSR loci are presented in Table 2. The number of pea aphids successfully genotyped ranged from 9 to 21 (average 15.211). The number of SSR alleles per locus was 1 to 12, with an average of 5.346. Only four SSR loci failed to show polymorphism among the samples of five geographical populations (Table 2), indicating that over 92% of loci are polymorphic. The average PIC in 52 loci is 0.575. The PIC of 84.6% (44) and 73.1% (38) loci exceeded 0.25 (reasonably informative) and 0.5 (highly informative), respectively (Table 2). The PIC values (0–0.25) in the seven types of motifs were under 30% apart from AAT/ATT and CCG/CGG. The percentage of most SSR motifs’ PIC, which exceeded 0.5, was not less than 50% (Figure 3). There is no significant difference in the PIC ratio between dinucleotide and trinucleotide SSRs.

## 4. Discussion

With the rapid increase in popularity of NGS, an increasing number of transcriptomes are being uploaded on public databases [48,49], signifying that the method using RNA-seq data to excavate SSR polymorphism information will acquire more data support and can be applied to additional species. This method contained complete flows for analysis of transcriptome data, providing an effective path to reduce the workload of biologist. The classical biology experiments to excavate polymorphic SSR loci, such as magnetic beads enrichment and 5′ anchored PCR method, are not easy and inevitably require large workloads and high costs [50,51]. The new idea, that researchers directly obtain polymorphic information from RNA-seq data, may bring about significant progress in the study of SSRs.

For the sake of controlling cost and workload, researchers were previously prone to using SSRs with more repeats because of the probability of SSR loci with fewer repeats showing high polymorphism might be unsatisfactory, resulting in a number of SSRs being ignored [10]. The proposed method directly excavates the polymorphism details to avoid the loss. During the test on pea aphids, the repeated number of SSR motifs in outputs from PSSRdt was not emphasized. Among the 52 loci tested, the repeat number of nine dinucleotide SSR motifs was entirely less than 12, and eleven trinucleotide repeats were all below 10 in the transcript set, and most of those loci were polymorphic (8, 88.9%; 11, 100%). There were six (66.7%) and seven (63.6%) dinucleotide and trinucleotide SSRs that had a PIC over 0.5. It is believed that researchers could acquire more polymorphic SSR loci for the analysis of population genetics when they adopt this method on the basis of those particular results. In addition, multiple sequences at the same SSR loci can be extracted from the sets of diverse transcriptomes, which provides more complete sequences for the design of PCR primers and reduces the impact of assembly errors.

PSSRdt can rapidly complete microsatellite detection and produce two different files, thereby helping users manage different issues and simplify workload. However, if part of the flanking sequences at the same locus of SSRs is mutated, the microsatellite loci with mutations will not be matched with others. Thus, missing a few potential polymorphic loci is unavoidable. Besides, the principle of the program regarding the identification of SSRs is based on whether the number of tandem repeats above certain thresholds; therefore, it is unable to distinguish between perfect and imperfect SSRs. Fortunately, the minimum repeat time of the imperfect SSRs is very close to or not lower than the thresholds [52,53], thereby leaving the imperfect loci generally unburied.

Although, transcriptome assembly is a complex task and has certain requirements for server hardware. In this study, we downloaded 44 pea aphid transcriptomes submitted in SRA database of *A. pisum*, and analyzed the backgrounds of RNA-seq data, including the experimental objectives and methods, the attributes of the samples, and the submitted institutions. In fact, there is no need to download and assemble all transcriptomes of research objects when the amount of data is abundant. Many cDNA libraries for RNA-seq have multiple duplications, and researchers can choose part of the raw data to save time. In addition, many factors can generate a large influence on microsatellite alteration, such as long-term pesticide treatment and extreme temperature. Thus, using more representative data that samples through different types of treatments can improve the possibility of polymorphic loci mining.

Many new polymorphic SSR loci were obtained in pea aphids and the subsequent experiment verified the efficiency of the method. This approach can be used for more species with sufficient transcriptome data. Numerous new SSR loci with polymorphisms in various species will be found and some research concerning microsatellites may be extended with the data support, for instance, the distribution rules and structural features of SSRs with polymorphisms in functional regions of genes, the influences of external environment on SSR mutations, and characteristics of SSR alleles among different species [54,55,56]. Moreover, examining the vast new loci among diverse species might be valuable to researchers studying the laws of SSR mutations during biological evolution [57].

## 5. Conclusions

In this study, a novel software and method were presented to efficiently excavate polymorphic SSR loci from RNA-seq data and tested on *A. pisum.* This concise method includes three stages: raw data processing, program development and application, and loci extraction and verification. The method provides a clear range for polymorphic loci mining and the experiment success rate was high compared with the traditional methods using RNA-seq data. PSSRdt was especially designed for SSR detection, which was better than the indel analysis software for SSR studies. The novel method provides a new path for rapidly screening numerous polymorphic SSR loci and abundant data for further studies of SSRs.

## Figures and Tables

**Table 1:** Summary of transcriptome assembly and simple sequence repeat (SSR) analysis.

Accession ID	Total Sequences ^a^	Total Size (bp)	Sequences with SSRs ^b^	Total SSRs	1 ^c^	2	3	4	5	6	Submission Institution
SRR063706	1584,7	2811,5408	7193	1499,2	9979	1860	3011	102	31	9	The University of Arizona
SRR063707	2667,3	9125,155	3736	5370	2947	1034	1351	21	14	3	The University of Arizona
SRR064408	8548	3035,495	1493	2138	985	508	630	9	4	2	Yale University
SRR064409	2422,2	3274,3820	7230	1175,3	6877	1456	3337	63	13	7	Yale University
SRR071347	9558	3108,732	1196	1653	663	300	680	6	2	2	Baylor College of Medicine
SRR073136	1014,5	3367,825	1481	2082	1122	376	574	7	2	1	University of Nebraska-Lincoln
SRR073272	1888,5	2141,4328	5184	8737	5423	1090	2153	52	13	6	University of Nebraska-Lincoln
SRR073274	7634	2076,016	407	514	151	73	283	6	1	0	University of Nebraska-Lincoln
SRR073276	3775,9	1020,0134	2122	2766	1143	375	1223	16	5	4	University of Nebraska-Lincoln
SRR353539	2789,8	4192,9144	1014,8	2241,8	1363,7	2943	5646	143	40	9	University of Nebraska-Lincoln
SRR073426	4643,7	1260,2772	2662	3487	1597	452	1410	19	5	4	Cornell university
SRR073573	2073,0	1504,9227	3212	4541	2745	593	1171	18	10	4	National Institute for Basic Biology
SRR073574	2164,6	8890,341	2478	3269	1405	447	1386	22	5	4	National Institute for Basic Biology
SRR073575	2001,8	2192,9991	4421	7262	3453	997	2758	40	9	5	National Institute for Basic Biology
SRR073576	1979,1	1799,6584	3611	5772	2199	872	2646	40	10	5	National Institute for Basic Biology
SRR073588	1668,3	2225,0609	6410	1291,7	7607	1742	3472	73	20	3	National Institute for Basic Biology
SRR074231	2336,2	4099,6212	1021,9	2242,0	1476,3	2745	4715	148	41	8	University of Nebraska-Lincoln
SRR074233	2133,8	2352,6029	7417	1472,3	9237	1897	3463	93	28	5	University of Nebraska-Lincoln
SRR075802	2376,3	3008,1669	8363	1792,7	1085,3	2391	4544	108	25	6	INRA ^d^
SRR075803	3108,7	3924,7189	1047,2	2068,1	1362,0	2470	4414	127	43	7	INRA
SRR097896	3299,3	3999,7626	9993	1783,0	1139,9	2139	4150	97	36	9	Centro Nacional de Análisis Genómico
SRR098330	3110,8	3628,8769	9981	1905,8	1276,0	2254	3898	104	35	7	Centro Nacional de Análisis Genómico
SRR1239439	3280,9	3742,6415	1015,9	1920,7	1267,7	2272	4090	119	40	9	Gene Expression Omnibus
SRR1239440	2037,3	3664,0425	7828	1561,6	8987	2073	4421	103	24	8	Gene Expression Omnibus
SRR1239441	1687,1	1151,2399	2089	2859	1654	365	820	15	2	3	Gene Expression Omnibus
SRR1239442	1581,1	2480,9531	6033	1218,1	7532	1470	3084	74	16	5	Gene Expression Omnibus
SRR1239443	1571,6	2352,5854	6212	1250,6	7943	1556	2918	68	18	3	Gene Expression Omnibus
SRR1239444	1232,7	1797,2772	4418	7931	5175	913	1783	45	11	4	Gene Expression Omnibus
SRR1239445	1376,8	2219,2206	5276	9848	6445	1139	2186	63	10	5	Gene Expression Omnibus
SRR1239446	6832,1	2486,0272	7185	1092,4	6648	1428	2759	68	15	6	Gene Expression Omnibus
SRR1239448	6799,5	2526,1149	7454	1140,1	7018	1473	2821	66	16	7	Gene Expression Omnibus
SRR1239449	2097,3	2681,8239	5932	9427	5914	1066	2369	58	13	7	Gene Expression Omnibus
SRR1239450	6334,7	1765,8260	3957	5229	2464	770	1954	28	9	4	Gene Expression Omnibus
SRR1239451	3222,4	8346,763	1515	1913	786	256	856	10	3	2	Gene Expression Omnibus
SRR1239452	2073,0	15049,227	3212	4541	2745	593	1171	18	10	4	Gene Expression Omnibus
SRR1239453	2080,9	8469,795	2382	3158	1270	441	1416	23	4	4	Gene Expression Omnibus
SRR1793299	2384,4	4240,2758	1037,1	2327,6	1495,4	2898	5216	155	39	14	Cornell university
SRR1793300	2042,4	3121,8422	7938	1492,9	9771	1845	3179	100	27	7	Cornell university
SRR924106	3001,7	3667,7810	9772	1859,3	1207,1	2275	4082	115	44	6	INRA
SRR924118	2568,1	3458,2798	8421	1534,3	9796	1877	3552	84	26	8	INRA
SRR924119	2478,0	3590,0468	8810	1695,9	1087,9	2045	3888	108	31	8	INRA
SRR924120	1589,6	2747,1266	5755	1011,2	6294	1202	2535	63	11	7	INRA
SRR924121	1600,2	2609,7341	6519	1377,2	8406	1733	3511	93	23	6	INRA
SRR924122	1445,5	2071,9694	5758	1126,0	7336	1348	2497	60	16	3	INRA

^a^ The number of transcripts assembled by Trinity; ^b^ The number of sequences containing SSRs; ^c^ Mononucleotide SSRs; ^d^ French National Institute for Agricultural Research.

**Table 2:** Polymorphism analysis of 52 microsatellite loci in pea aphid individuals.

Locus	N	N_A_	F_M_	PIC
3	12	5	0.4167	0.5748
4	18	6	0.5278	0.6194
5	20	7	0.4250	0.7164
6	18	6	0.3333	0.7444
7	14	3	0.6786	0.4090
8	17	9	0.2059	0.8313
9	21	6	0.4286	0.7006
10	12	6	0.2917	0.7517
13	16	9	0.2813	0.8122
14	18	8	0.4444	0.7118
15	16	1	1.0000	0.0000
16	16	7	0.4375	0.7081
17	15	3	0.8333	0.2604
18	21	8	0.2381	0.8207
19	14	5	0.4643	0.6469
21	16	2	0.6250	0.3589
22	11	5	0.5000	0.6257
23	11	6	0.3182	0.7436
27	17	4	0.6176	0.5239
29	13	6	0.3462	0.6874
31	11	11	0.2273	0.8595
33	16	2	0.9375	0.1103
34	12	3	0.4583	0.5697
35	18	4	0.4722	0.5851
38	17	4	0.6176	0.5269
39	17	8	0.2647	0.7888
40	20	1	1.0000	0.0000
41	10	7	0.3500	0.7700
43	12	8	0.2917	0.8013
46	17	5	0.3235	0.7130
47	16	1	1.0000	0.0000
48	13	7	0.4231	0.6867
49	16	2	0.8750	0.1948
51	14	5	0.3214	0.7248
52	20	4	0.4500	0.5249
53	14	6	0.3929	0.7072
101	15	3	0.7667	0.3227
102	15	12	0.2000	0.8685
108	12	7	0.2917	0.7614
109	16	3	0.8438	0.2478
110	15	6	0.4333	0.6675
112	17	9	0.2353	0.8319
113	15	3	0.8333	0.2710
114	15	9	0.3667	0.7762
116	9	3	0.7222	0.3709
117	9	7	0.2778	0.8053
119	12	8	0.2500	0.7957
121	18	5	0.2778	0.7429
122	17	2	0.8824	0.1861
128	11	5	0.3182	0.7319
131	18	5	0.3056	0.7165
132	18	1	1.0000	0.0000
Mean	15.2115	5.3462	0.4966	0.5751

N, Number of aphids successfully genotyped; N_A_, Number of alleles per locus; F_M_, Frequency of major allele; PIC, Polymorphism information content.

## Supplementary Materials

The following are available online at https://www.mdpi.com/2073-4425/10/11/917/s1, Table S1: The criteria for SSR detection, Table S2: Statistics of RNA-seq raw reads and clean reads after data filtering, Table S3: Characteristics of 52 microsatellite loci developed for *A. pisum*, File S1 and File S2 are the two output files generated by PSSRdt (findStr.result and findStr.result.detail).
