# Supplementary material for: A Novel Software and Method for the Efficient Development of Polymorphic SSR Loci Based on Transcriptome Data
Source: Genes (Basel). 2019 Nov 11;10(11):917. doi: 10.3390/genes10110917 (PMC6895799; doi:10.3390/genes10110917)
Supplement: Supplementary file 1 [file genes-10-00917-s001.zip › Supplementary Materials/Supplementary Table 1.docx]

**Supplementary Table 1. The criteria for SSR detection.**

| **Motif size** | **Minimum repeat times** |
| --- | --- |
| 1 | 10 |
| 2 | 6 |
| 3 | 5 |
| 4 | 5 |
| 5 | 5 |
| 6 | 5 |
