# Supplementary material for: A Novel Software and Method for the Efficient Development of Polymorphic SSR Loci Based on Transcriptome Data
Source: Genes (Basel). 2019 Nov 11;10(11):917. doi: 10.3390/genes10110917 (PMC6895799; doi:10.3390/genes10110917)
Supplement: Supplementary file 1 [file genes-10-00917-s001.zip › Supplementary Materials/Supplementary Table 2.docx]

**Supplementary Table 2. Statistics of RNA-seq raw reads and clean reads after data filtering.**

| **Accession ID** | **Raw reads** | | **Clean reads** | |
| --- | --- | --- | --- | --- |
|  | **Total number** | **GC%** | **Total number** | **GC%** |
| SRR063706 | 27747135 | 42 | 27420552 | 42 |
| SRR063707 | 28775664 | 42 | 28440636 | 42 |
| SRR064408 | 24521218 | 38 | 23459037 | 38 |
| SRR064409 | 26243930 | 40 | 25427879 | 40 |
| SRR071347 | 54565860 | 39 | 37594402 | 36 |
| SRR073136 | 7006420 | 47 | 5856215 | 46 |
| SRR073272 | 6355021 | 50 | 5354621 | 49 |
| SRR073274 | 5189687 | 51 | 4325212 | 49 |
| SRR073276 | 3387696 | 48 | 2829317 | 48 |
| SRR353539 | 5927088 | 43 | 5155080 | 43 |
| SRR073426 | 4726216 | 38 | 4606013 | 38 |
| SRR073573 | 12212938 | 44 | 10896803 | 44 |
| SRR073574 | 5236662 | 46 | 4763327 | 45 |
| SRR073575 | 8369405 | 44 | 7938926 | 44 |
| SRR073576 | 5668342 | 45 | 4713998 | 44 |
| SRR073588 | 16456663 | 44 | 13403398 | 42 |
| SRR074231 | 8869333 | 43 | 7604430 | 43 |
| SRR074233 | 6384100 | 40 | 5525365 | 40 |
| SRR075802 | 23557140 | 51 | 8467210 | 45 |
| SRR075803 | 23264412 | 53 | 6887168 | 46 |
| SRR097896 | 111779326 | 43 | 75114338 | 41 |
| SRR098330 | 126263308 | 50 | 69344438 | 47 |
| SRR1239439 | 93097593 | 36 | 92163842 | 35 |
| SRR1239440 | 101313506 | 40 | 100122356 | 40 |
| SRR1239441 | 91946734 | 36 | 91079588 | 35 |
| SRR1239442 | 109616712 | 33 | 108646204 | 33 |
| SRR1239443 | 82776192 | 39 | 81963105 | 39 |
| SRR1239444 | 102979257 | 40 | 102163410 | 40 |
| SRR1239445 | 106808567 | 39 | 105838150 | 39 |
| SRR1239446 | 80133908 | 36 | 79556386 | 36 |
| SRR1239448 | 100710783 | 35 | 99737151 | 35 |
| SRR1239449 | 94077934 | 39 | 92937535 | 39 |
| SRR1239450 | 106615474 | 40 | 105773532 | 40 |
| SRR1239451 | 82734259 | 34 | 82005069 | 33 |
| SRR1239452 | 71796221 | 35 | 71062677 | 35 |
| SRR1239453 | 90793111 | 38 | 90186605 | 37 |
| SRR1793299 | 28088323 | 40 | 25067173 | 40 |
| SRR1793300 | 31373858 | 40 | 27871070 | 40 |
| SRR924106 | 45969742 | 35 | 42600208 | 34 |
| SRR924118 | 34908308 | 36 | 32356726 | 35 |
| SRR924119 | 45817756 | 35 | 42460112 | 34 |
| SRR924120 | 37385518 | 34 | 34697926 | 33 |
| SRR924121 | 25848748 | 35 | 23839724 | 34 |
| SRR924122 | 34402416 | 36 | 31922552 | 35 |

**Supplementary Table 3. Characteristics of 52 microsatellite loci developed for *A. pisum.***

| Locus | Primer (5'-3')  Forward Reverse | | SSR motif | Ta(℃) |
| --- | --- | --- | --- | --- |
| 3 | GATTAACTGTGAGGAAACA | CGTATCTCACGACCAC | (AC） | 56 |
| 4 | TGTCTGCGAGCATTGGC | GACGACCGAGGAGGAAA | (AC） | 58 |
| 5 | CGGAAGAGTGCAACAAT | TCGTCCCTCTAATCGTG | (CA） | 60 |
| 6 | CTACGACACCACCACAGCA | CAAAGAGGCAAAGGCAA | (AC） | 60 |
| 7 | GCTGACCGACTCACATA | CAAACTTACACGGGATC | (GT） | 60 |
| 8 | TACGGGTCACCTGAAGA | TAAGCTGCTGCGAACAC | (TG） | 57 |
| 9 | GACGACCACGACAACGA | ACGACACGACGGCAGAG | (CT） | 59 |
| 10 | CAATGCCAAGGTTAGATGC | ATGCGGAGTGGTGAGGG | (CT） | 57 |
| 13 | TGTTTTATGAGTCAATGCTA | ATTTACAAAGATCCAGGC | (GTA） | 54 |
| 14 | ACCAACGGACGGTAGACA | CGAATGCAATTTATCATTTAGG | (AAT） | 60 |
| 15 | CAGGAACGGAAAACGATT | CCCTGTCTGCGTATGCTA | (TAA） | 58 |
| 16 | ACATTCGCTATCGGGAGTC | CCGTAGTAGGAAGGCAAC | (ACA） | 56 |
| 17 | CGTGCAGTTTAAATGCATC | TTCGTATCGGCAATGGTA | (ATC） | 59 |
| 18 | GGACGACGACCACGACA | ACGACACGACGGCAGAG | (CT） | 56 |
| 19 | ATAACACCACCGTCCCA | GTCATAATGAATTTCTCATCGTA | (AT） | 57 |
| 21 | AGCAGCAGCTTCCAAAC | GCAGCGAATACGGTCAT | (AT） | 60 |
| 22 | CACCTGCTCACCCACTG | ACAAACGAACCTACAACAAAT | (AT） | 56 |
| 23 | TAAGTATCGAAACTCTAACC | CGAGATTCTTCCCATTA | (AT） | 56 |
| 27 | ACCGTTGTAGTCGAAAGC | CAAACCATCATCCGTAGC | (TTG） | 56 |
| 29 | TACAATGCCACAAGTGC | TAGCACCATCTAAGTGAATA | (AT） | 57 |
| 31 | GGTATACATCGGCAAGC | GTACAAATGGATGGATAATAAA | (AC） | 56 |
| 33 | GAAGTTGGCTGTCTTGA | ATTTGCTGCACTTCTTG | (AT） | 57 |
| 34 | AACGACGGGAACGACTA | GACGGAACTTTATCATTACTTG | (AT） | 60 |
| 35 | GCCTTGCTGGATGGACG | TGCGGAGCTGCTCTGGT | (TGC） | 59 |
| 38 | TCGTTCCGATTGAGTGT | TCGAACAATCGAAATCC | (TAT） | 58 |
| 39 | GATGGAATGCGGCTCGTA | TGGCTCATCGCTGTTCG | (GT） | 58 |
| 40 | AATCCTTTCTTTCTAATACC | AGACAAAGCGATCTCAA | (ATA） | 59 |
| 41 | ACCGTTGTAGTCGAAAGC | CAAACCATCATCCGTAGC | (TTG） | 56 |
| 43 | TGCGTGTTATTTACGATTTG | GTTGTGCCCGTTACCCT | (TG） | 54 |
| 46 | ACCTACTTGTGGTTTTC | ACCCATATTATGGACAT | (AT） | 57 |
| 47 | CACAAACTAGGTATGTACGGAA | CACCAGCGGCATTTTAT | (AAT） | 57 |
| 48 | TTTGAAATAACTAATCTACCCTG | ATGGGAACCACTGTATGAC | (TG） | 60 |
| 49 | CGGTGAATAACAAACAG | CTGCGACAACAACCATA | (AAT） | 60 |
| 51 | TAGTACGACCTTCTTGGG | GACAGTCCCTTCTACGC | (GT） | 62 |
| 52 | TTCGTCGTTTCAGTCAG | TAAGGGTAAATAGTTGGTT | (AC） | 56 |
| 53 | GTAGCGTCATAATAGTCCG | CCGTGTTAAAGCTTCCA | (CA） | 59 |
| 101 | GGCGGAAACTCTTGATA | TGGACTCGCTCGACCTG | (AT） | 60 |
| 102 | ATTCGTTTCAATAAGCA | TTTTGTCAGCAATCCTT | (AT） | 55 |
| 108 | AGATTATGCGTAGGATT | AAAATGAGATCGCAGTA | (CT） | 60 |
| 109 | TCAAACCGGAAATAACA | ACTCAACCGAAAATGTG | (TGA） | 55 |
| 110 | CACTACATGATTCCCATAC | CAGGCAATTCATCAGTAA | (TGA） | 54 |
| 112 | AGCCTCCTCTGCTGTGA | CGCCGAAACGAAACTAG | (GTA） | 58 |
| 113 | TAGCTCAAGTGCTGGGTG | ATGCCTGTCATCTTCGTT | (AGT） | 56 |
| 114 | CAACCGACGATGATGTA | AGGGTCTGAGGCTTCTA | (AGT） | 59 |
| 116 | GTTGTAACCGTGATTCTGCT | AGGTCGCACTACGCACA | (CTG） | 53 |
| 117 | AATAACGGTTGTTGTGG | TCATTTAGCGGATAGGT | (TG） | 61 |
| 119 | ATGATCGGTGATGAGTG | ATACCCTTTGTCTGTGC | (TG） | 58 |
| 121 | CAATAACTCGTCGCCTCCC | TCGTCGGTCCACTTTGGTT | (CCG） | 59 |
| 122 | ACGGAAAGTCTTCACCAACG | CGATTCGGTTTCGGGAGT | (GCC） | 60 |
| 128 | ATAGGTGGACGTATGGG | GTCTAGCCGGATGTTGT | (GAG） | 54 |
| 131 | GGAAGAAATACGCACTACGC | GCTGCCGACACGGAAAT | (AC） | 58 |
| 132 | TTCGTCGTTTCAGTCAG | TAAGGGTAAATAGTTGGTT | (AC） | 58 |
