# Supplementary material for: A Novel Software and Method for the Efficient Development of Polymorphic SSR Loci Based on Transcriptome Data
Source: Genes (Basel). 2019 Nov 11;10(11):917. doi: 10.3390/genes10110917 (PMC6895799; doi:10.3390/genes10110917)
Supplement: Supplementary file 1 [file genes-10-00917-s001.zip › Supplementary Materials/Supplementary Table 3.docx]

**Supplementary Table 3. Characteristics of 52 microsatellite loci *developed for*** ***Acyrthosiphon pisum.***

| Locus | Primer (5'-3')  Forward Reverse | | SSR motif | Ta(℃) |
| --- | --- | --- | --- | --- |
| 3 | GATTAACTGTGAGGAAACA | CGTATCTCACGACCAC | (AC） | 56 |
| 4 | TGTCTGCGAGCATTGGC | GACGACCGAGGAGGAAA | (AC） | 58 |
| 5 | CGGAAGAGTGCAACAAT | TCGTCCCTCTAATCGTG | (CA） | 60 |
| 6 | CTACGACACCACCACAGCA | CAAAGAGGCAAAGGCAA | (AC） | 60 |
| 7 | GCTGACCGACTCACATA | CAAACTTACACGGGATC | (GT） | 60 |
| 8 | TACGGGTCACCTGAAGA | TAAGCTGCTGCGAACAC | (TG） | 57 |
| 9 | GACGACCACGACAACGA | ACGACACGACGGCAGAG | (CT） | 59 |
| 10 | CAATGCCAAGGTTAGATGC | ATGCGGAGTGGTGAGGG | (CT） | 57 |
| 13 | TGTTTTATGAGTCAATGCTA | ATTTACAAAGATCCAGGC | (GTA） | 54 |
| 14 | ACCAACGGACGGTAGACA | CGAATGCAATTTATCATTTAGG | (AAT） | 60 |
| 15 | CAGGAACGGAAAACGATT | CCCTGTCTGCGTATGCTA | (TAA） | 58 |
| 16 | ACATTCGCTATCGGGAGTC | CCGTAGTAGGAAGGCAAC | (ACA） | 56 |
| 17 | CGTGCAGTTTAAATGCATC | TTCGTATCGGCAATGGTA | (ATC） | 59 |
| 18 | GGACGACGACCACGACA | ACGACACGACGGCAGAG | (CT） | 56 |
| 19 | ATAACACCACCGTCCCA | GTCATAATGAATTTCTCATCGTA | (AT） | 57 |
| 21 | AGCAGCAGCTTCCAAAC | GCAGCGAATACGGTCAT | (AT） | 60 |
| 22 | CACCTGCTCACCCACTG | ACAAACGAACCTACAACAAAT | (AT） | 56 |
| 23 | TAAGTATCGAAACTCTAACC | CGAGATTCTTCCCATTA | (AT） | 56 |
| 27 | ACCGTTGTAGTCGAAAGC | CAAACCATCATCCGTAGC | (TTG） | 56 |
| 29 | TACAATGCCACAAGTGC | TAGCACCATCTAAGTGAATA | (AT） | 57 |
| 31 | GGTATACATCGGCAAGC | GTACAAATGGATGGATAATAAA | (AC） | 56 |
| 33 | GAAGTTGGCTGTCTTGA | ATTTGCTGCACTTCTTG | (AT） | 57 |
| 34 | AACGACGGGAACGACTA | GACGGAACTTTATCATTACTTG | (AT） | 60 |
| 35 | GCCTTGCTGGATGGACG | TGCGGAGCTGCTCTGGT | (TGC） | 59 |
| 38 | TCGTTCCGATTGAGTGT | TCGAACAATCGAAATCC | (TAT） | 58 |
| 39 | GATGGAATGCGGCTCGTA | TGGCTCATCGCTGTTCG | (GT） | 58 |
| 40 | AATCCTTTCTTTCTAATACC | AGACAAAGCGATCTCAA | (ATA） | 59 |
| 41 | ACCGTTGTAGTCGAAAGC | CAAACCATCATCCGTAGC | (TTG） | 56 |
| 43 | TGCGTGTTATTTACGATTTG | GTTGTGCCCGTTACCCT | (TG） | 54 |
| 46 | ACCTACTTGTGGTTTTC | ACCCATATTATGGACAT | (AT） | 57 |
| 47 | CACAAACTAGGTATGTACGGAA | CACCAGCGGCATTTTAT | (AAT） | 57 |
| 48 | TTTGAAATAACTAATCTACCCTG | ATGGGAACCACTGTATGAC | (TG） | 60 |
| 49 | CGGTGAATAACAAACAG | CTGCGACAACAACCATA | (AAT） | 60 |
| 51 | TAGTACGACCTTCTTGGG | GACAGTCCCTTCTACGC | (GT） | 62 |
| 52 | TTCGTCGTTTCAGTCAG | TAAGGGTAAATAGTTGGTT | (AC） | 56 |
| 53 | GTAGCGTCATAATAGTCCG | CCGTGTTAAAGCTTCCA | (CA） | 59 |
| 101 | GGCGGAAACTCTTGATA | TGGACTCGCTCGACCTG | (AT） | 60 |
| 102 | ATTCGTTTCAATAAGCA | TTTTGTCAGCAATCCTT | (AT） | 55 |
| 108 | AGATTATGCGTAGGATT | AAAATGAGATCGCAGTA | (CT） | 60 |
| 109 | TCAAACCGGAAATAACA | ACTCAACCGAAAATGTG | (TGA） | 55 |
| 110 | CACTACATGATTCCCATAC | CAGGCAATTCATCAGTAA | (TGA） | 54 |
| 112 | AGCCTCCTCTGCTGTGA | CGCCGAAACGAAACTAG | (GTA） | 58 |
| 113 | TAGCTCAAGTGCTGGGTG | ATGCCTGTCATCTTCGTT | (AGT） | 56 |
| 114 | CAACCGACGATGATGTA | AGGGTCTGAGGCTTCTA | (AGT） | 59 |
| 116 | GTTGTAACCGTGATTCTGCT | AGGTCGCACTACGCACA | (CTG） | 53 |
| 117 | AATAACGGTTGTTGTGG | TCATTTAGCGGATAGGT | (TG） | 61 |
| 119 | ATGATCGGTGATGAGTG | ATACCCTTTGTCTGTGC | (TG） | 58 |
| 121 | CAATAACTCGTCGCCTCCC | TCGTCGGTCCACTTTGGTT | (CCG） | 59 |
| 122 | ACGGAAAGTCTTCACCAACG | CGATTCGGTTTCGGGAGT | (GCC） | 60 |
| 128 | ATAGGTGGACGTATGGG | GTCTAGCCGGATGTTGT | (GAG） | 54 |
| 131 | GGAAGAAATACGCACTACGC | GCTGCCGACACGGAAAT | (AC） | 58 |
| 132 | TTCGTCGTTTCAGTCAG | TAAGGGTAAATAGTTGGTT | (AC） | 58 |
